# Eco-friendly pomegranate production: Balancing energy consumption and environmental impact

**DOI:** 10.1371/journal.pone.0329204

**Published:** 2025-08-13

**Authors:** Amir Azizpanah, Mohammad Salavrzi Zadeh, Alaa Kamil Abed, Morteza Taki

**Affiliations:** 1 Department of mechanics Biosystem, College of Agriculture, Ilam University, Ilam, Iran; 2 Department of Architectural Engineering, Ilam University, Ilam, Iran; 3 Department of Biosystems Mechanical Engineering, Design and Manufacturing, College of Agriculture, Tarbiat Modares University, Iran; 4 Department of Agricultural Machinery and Mechanization Engineering, Faculty of Agricultural Engineering and Rural Development, Agricultural Sciences and Natural Resources University of Khuzestan, Mollasani, Iran; Nuclear Science and Technology Research Institute, IRAN, ISLAMIC REPUBLIC OF

## Abstract

Pomegranate production in Siab (Lorestan), Iran, faces significant challenges related to high energy consumption and environmental degradation, particularly due to inefficient use of agricultural inputs such as fertilizers, water and machinery. These inefficiencies contribute to increased greenhouse gas emissions and higher production costs, making optimization efforts essential for sustainable development. This study investigated the optimization of energy consumption and the reduction of environmental impacts in pomegranate production using a combination of Data Envelopment Analysis (DEA) and Life Cycle Assessment (LCA). Data were collected through interviews with farmers and agricultural experts in the region, supported by structured questionnaires. The research evaluated several energy indicators, including an energy ratio of 2.14, which indicates that every unit of energy input yields more than double in output—comparable to other fruit crops like apple or citrus, which typically range between 1.5 and 3.0. Energy productivity was found to be 1.12 kgMJ^-1^, meaning 1.12 kilograms of pomegranate are produced per megajoule of energy consumed, while specific energy was calculated at 0.89 MJkg ⁻ ¹, showing relatively efficient energy use compared to similar horticultural crops. Net energy gain was 17,142.33 MJha ⁻ ¹, with total energy consumption at 15,211.04 MJha ⁻ ¹ and an energy output of 32,353.38 MJha ⁻ ¹. Economic analysis revealed a gross value of 9,081.64 USDha ⁻ ¹, fixed costs of 204.44 USDha ⁻ ¹, and gross revenue of 8,059.42 USDha ⁻ ¹, resulting in a benefit-to-cost ratio of 0.83. LCA results showed that optimized practices significantly reduced environmental impacts across most of the 15 intermediate environmental indicators analyzed. For instance, global warming potential was reduced from 40.563 kg CO₂ eq per ton of pomegranate under conventional methods to 35.975 kg CO₂ eq with optimized practices. DEA under the Variable Returns to Scale (VRS) model revealed that 66.68% of the surveyed orchards operated at 100% technical efficiency. The average technical efficiency across all units was estimated at 98.96%. The remaining 33.32% of orchards were identified as technically inefficient. Scale efficiency averaged at 99.39%, suggesting that most farms operate near optimal size.

## Introduction

Pomegranate (*Punica granatum* L.), a significant horticultural product in Iran, is a small tree cultivated in semi-arid and Mediterranean regions and is renowned as one of the most famous fruits grown in Western Asia and the Middle East [1]. Iran, the origin of the pomegranate, leads the world in pomegranate diversity and quality and ranks third globally, after India and China, in terms of cultivation area and production rate [2]. The total cultivation area for this fruit tree in Iran is approximately 60,000 hectares. Lorestan province, with a cultivation area of 3,163 hectares and a total production of 59,562 tons, ranks tenth in the country.

Currently, ensuring food security for the world’s growing population without compromising essential land and water resources [3], and with minimal environmental impact, has become a significant challenge for sustainable agriculture [4,5]. Two major challenges currently under scrutiny are the optimization of energy consumption and the reduction of Greenhouse Gas (GHG) [6,7] emissions in the agricultural sector, along with the economic implications of input usage [8,9]. Given global concerns over fossil fuel depletion and climate change [10], precise planning of input use has become critical across agricultural systems [11,12]. According to the FAO (2019) [13], increased energy use in agriculture has boosted productivity and rural economic growth [14]; however, intensive farming practices reliant on chemical inputs, machinery, and fossil fuels have significantly contributed to GHG emissions. This shift underscores the need to examine how energy use in agriculture translates into environmental impacts [15]. Generally, energy consumption and GHG emission patterns in agricultural systems depend on factors such as farm size [16], technology [17], input application rates, diesel consumption, and crop yields [18,19]. Troujeni et al (2018) [20], analyzed the energy use patterns and economic aspects of pomegranate production in Behshahr, Iran. Data from 83 orchards revealed a total energy input of 11,195.06 MJha^-1^, with diesel fuel (45.81%) and chemical fertilizers (23.47%) as the main contributors. Energy use efficiency was 1.18, indicating a positive net energy gain. Sensitivity analysis showed that increasing irrigation water and chemical fertilizer energy inputs boosted yield, while diesel fuel and machinery had negative impacts. The benefit-to-cost ratio was 5.57, demonstrating profitable production despite relatively low yields compared to other regions. Behrooznia et al (2024) [21], presented a comprehensive analysis of energy use patterns and economic aspects of pomegranate production in Behshahr, Iran. The total energy input was calculated to be 11,195.06 MJha^-1^, dominated by diesel fuel (45.81%) and chemical fertilizers (23.47%), indicating a heavy reliance on non-renewable energy sources. Energy use efficiency was found to be 1.18, suggesting a positive net energy gain, while the benefit-to-cost ratio stood at 5.57, highlighting the profitability of pomegranate cultivation despite relatively low yields compared to other regions. Sensitivity analysis revealed that irrigation water and chemical fertilizer inputs positively influenced yield, whereas diesel fuel and machinery had negative impacts, emphasizing the need for optimized resource management. These findings provide valuable insights for improving sustainability and economic returns in pomegranate farming through better energy use practices.

One effective approach to improving energy efficiency and reducing environmental burdens is through comprehensive energy flow analysis in crop production [22]. Understanding how various factors influence energy performance—while integrating technical, economic, and environmental dimensions—is crucial for optimizing agricultural practices [23,24].

In pomegranate orchards, similar to other high-input crops, significant amounts of energy—especially from fossil fuels—are consumed during production processes. The combustion of these fuels contributes directly to climate change by releasing CO₂ and other GHGs into the atmosphere, thereby posing risks to both ecosystems and human health [25,26]. Furthermore, the production and application of agrochemicals also entail indirect energy use and emissions, adding to the environmental footprint of pomegranate farming. Sustainable production frameworks emphasize that all agricultural activities must align with principles of ecosystem protection, resource conservation, and socio-economic development [27,28].

Despite increasing attention to sustainable practices in agriculture, pomegranate production in the Kuhdasht region of Lorestan province remains understudied, particularly regarding localized data on energy use and environmental impacts. Moreover, no prior study has applied an integrated Data Envelopment Analysis (DEA) with Life Cycle Assessment (LCA) approach to assess and optimize both efficiency and environmental outcomes in this context. Previous research has largely focused on broader national or international trends, leaving regional-specific inefficiencies and mitigation opportunities unexplored. This study addresses these gaps by providing empirical insights into energy flows, economic performance, and GHG emissions using a combined DEA and LCA framework. By doing so, it aims to offer actionable strategies for enhancing sustainability in pomegranate production, which may serve as a model for similar fruit crops in comparable agro-ecological zones.

## Materials and methods

### Study site

The research was conducted in Kuhdasht County, Lorestan Province, Iran. The study commenced in May 2024 with the distribution of questionnaires and concluded on July 25, 2024. Data analysis and finalization of the report were completed by September 2024. Data collection was carried out through face-to-face interviews with pomegranate farmers. This study involved gathering primary data directly from farmers, complemented by information extracted from existing literature. Informed consent was obtained from all participants prior to conducting the interviews. Farmers were provided with detailed information regarding the purpose, procedures, and their rights as participants. They were assured that their responses would remain confidential and had the option to withdraw at any time without consequence. Consent forms were signed by all participants, ensuring voluntary involvement and understanding of the study. It is important to note that this research did not involve minors or clinical trials.

To construct the sample frame, a complete list of registered pomegranate growers in Kuhdasht County was obtained from the local agricultural extension office. Using a random number generator, 21 farmers were selected from this list to participate in the study. The sample size (n = 21) was calculated based on Eq. (1) (Kaab et al., 2019), assuming a total population (N) of approximately 300 registered pomegranate farmers in the region, a 95% confidence level (Z = 1.96), a 50% response distribution (p = 0.5), and a margin of error (d) of ±5%.


$$n=\frac{\frac{z^{2}pq}{d^{2}}}{1+\frac{1}{N}(\frac{z^{2}pq}{d^{2}}-1)}$$ (1)

It should be noted that while this sample size meets the minimum requirements for statistical estimation under these assumptions, it may not fully represent the heterogeneity within the farming community due to limited variability in farm size, management practices, and resource use. The survey instrument used in this study was developed based on established frameworks used in similar agricultural energy studies [23]. Prior to full-scale implementation, the questionnaire underwent a pre-testing phase with a pilot group of five farmers who were not included in the final sample. Feedback from the pilot was used to refine the clarity and structure of the questions. Additionally, content validity was ensured through expert review by two agricultural engineers and one environmental scientist specializing in agro-energy systems. Internal consistency was evaluated using Cronbach’s alpha, which yielded a value of 0.82, indicating acceptable reliability.

Analysis of energy consumed for pomegranate production was conducted by quantifying the direct and indirect energy inputs used during the production cycle. Energy consumption was calculated using standard coefficients (Table 1), including human labor, agricultural machinery, diesel fuel, chemical fertilizers (P, N, and K), manure, water for irrigation, herbicides, electricity and yield. Table 2 presents the energy indicators used in this study, such as energy ratio, energy productivity, specific energy, and net energy gain [23].

**Table 1:** Energy equivalents of the inputs and outputs in pomegranate production.

Item	Unit	Energy equivalent (MJ unit^-1^)	Reference
Human labor	h	1.96	[30]
Agricultural machinery	h	62.7	[23]
Diesel fuel	L	56.31	[31]
Phosphorous (P_2_O_5_)	kg	12.44	[31]
Nitrogen	kg	66.14	[29]
Potassium(K_2_O)	kg	11.15	[29]
Manure	kg	0.3	[32]
Herbicide	L	238	[30]
Water for irrigation	M^3^	1.02	[30]
Electricity	KWh	11.93	[29]
Pomegranate	kg	2.4	[4]

**Table 2:** Energy indicators in the pomegranate production systems.

Index	Unit	Equation	Reference
Energy Ratio	–	$\frac{Output\;energy\;\left(MJ\;ha^{-1}\right)}{Total\;input\;energy\;\left(MJ\;ha^{-1}\right)}$	[23]
Energy productivity	KgMJ^-1^	$\frac{Pomegranate\;yield\;\left(kg\;ha^{-1}\right)}{Total\;input\;energy\;\left(MJ\;ha^{-1}\right)}$	[29]
Specific Energy	$MJKg^{-1}$	$\frac{Total\;input\;energy\;\left(MJ\;ha^{-1}\right)}{Pomegranate\;yield\;\left(kg\;ha^{-1}\right)}$	[18]
Net energy gain	MJha^-1^	$Output\;energy\;\left(MJ\;ha^{-1}\right)-Total\;input\;energy\;\left(MJ\;ha^{-1}\right)$	[4]

### Economic calculations and assessment

The economic indicators utilized in this research (Table 3) all expressed in terms of dollars per hectare ($ha^-1^). The cost of production is also calculated on $ha^-1^ [29]. In Table 3, GR is Gross Income ($ha^-1^), GVP is Gross Value Product ($ha^-1^), VCP is Variable Costs of Production ($ha^-1^), CY is Crop Yield (kgha^-1^), CP is Crop Price ($), NI is Net Income ($ha^-1^), TPC is Total Product Costs ($ha^-1^), FPC is Fixed Production Costs ($ha^-1^), PCR is Profit-Cost Ratio and P is Production efficiency (kg$^-1^).

**Table 3:** Economic indicators used for pomegranate production evaluation.

GR=GVP−VCP	(2)	GVP=CY×CP	(3)	NR=GVP−TC	(4)
GTPC=FPC+VCP	(5)	$PCR=\frac{VCP}{TPC}$	(6)	$P=\frac{CY\;}{TPC}\;$	(7)

### Environmental impact analysis by Life Cycle Assessement (LCA) method

The LCA method was employed to evaluate and compare the environmental impacts of pomegranate production. This method adheres to the ISO 14040 standards [33]. LCA provides a comprehensive approach to assessing the environmental effects of crop production, processes, and services by considering multiple dimensions and impact categories [31,34]. LCA is highly effective in studying the environmental impacts of products, processes, or activities by identifying and collecting data on resource use, emissions, and residues released into the environment [35,36]. In this research, LCA was applied to pomegranate production based on ISO 14040 standards, and the environmental impact data were analyzed using the SimaPro version 9.2.0.1 software package. The LCA methodology consists of four key steps: (i) defining the goal and scope; (ii) performing inventory analysis; (iii) evaluating life cycle impacts; and (iv) interpreting the results. The first step involved defining the general framework of the field operation, including the research goal, performance units, and system boundaries. The primary goal was to evaluate the potential environmental impact of pomegranate production in the Kuhdasht region. For this study, the performance unit was set at one ton of pomegranates, meaning that the environmental indicators were assessed based on this quantity [31]. The system boundary was defined as cradle-to-gate, encompassing all stages from raw material extraction, through agricultural operations, up to the point of fruit harvest (gate). This definition has been added to clarify the scope and ensure transparency. To complete the inventory analysis, both background and foreground data were collected. Background data, which relate to the environmental effects of each input, are available in the LCA databases within the SimaPro software. Foreground data, which pertain to the production process, were collected through questionnaires and face-to-face interviews with farmers. These data included the amounts of various inputs consumed in the pomegranate orchards, such as labor, agricultural machinery, diesel fuel, fertilizers, herbicides, and water for irrigation. By entering these data into the SimaPro software, their environmental effects were obtained as outputs [31,37].

**Fig 1:**
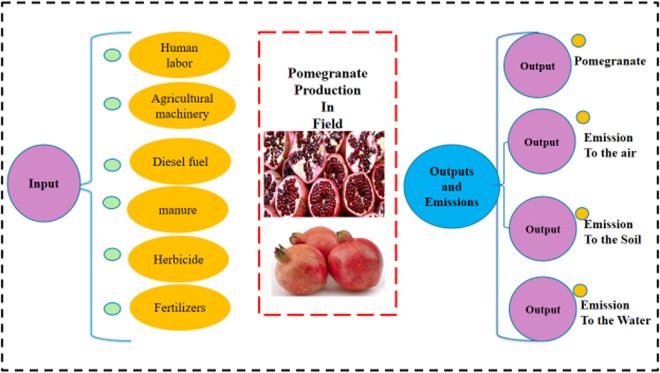
System boundary for pomegranate production in the study area.

The environmental impacts of pomegranate production in Kuhdasht County were analyzed across several categories, including human health (carcinogenic and non-carcinogenic effects), respiratory effects (both organic and inorganic), and ecological effects (terrestrial and aquatic ecotoxicity, acidification, and eutrophication). Additionally, the study assessed impacts on ionizing radiation, ozone layer depletion, land occupation, global warming potential, and the consumption of non-renewable energy and mineral resources. The assessment also accounted for direct emissions resulting from agricultural inputs such as diesel fuel, chemical fertilizers (phosphorus, potassium, nitrogen), and pesticides, which contribute to air, water, and soil pollution [33]. Various methods can be used for assessing environmental impacts through LCA, such as IMPACT 2002 + , ReCiPe 2016, Eco-indicator 99, CML 2001, EDIP 2003, and EPD [31]. This research adopted the IMPACT 2002 + method, which combines methodologies such as IPCC for global warming potential, CML Baseline 2001 for acidification and eutrophication, and Eco-indicator 99 for human toxicity categories [38]. This model was selected due to its comprehensive coverage of mid-point and end-point impact categories, allowing for a more integrated interpretation of environmental burdens. Table 4 shows the coefficients of air emissions due to diesel fuel consumption in pomegranate orchards. Fig 1 shows the system boundary for calculating the energy and environmental pollutants for pomegranate production in this study.

**Table 4:** Coefficients of air emissions due to diesel fuel consumption in pomegranate orchards.

Emissions due to diesel fuel to air	Value (KgMJ^-1^)	Unit
Carbon dioxide (CO_2_)	74.5	Kg
Sulfur dioxide (SO_2_)	2.41E-2	Kg
Methane (CH_4_)	3.08E-3	Kg
Benzene	1.74E-4	Kg
Cadmium (Cd)	2.39E-7	Kg
Chromium (Cr)	1.19E-6	Kg
Copper (Cu)	4.06E-5	Kg
Dinitrogen monoxide (N_2_O)	2.86E-3	Kg
Nickel (Ni)	1.67E-6	Kg
Zink (Zn)	2.39E-5	Kg
Benzo (a) pyrene	7.16E-7	Kg
Ammonia (NH_3_)	4.77E-4	Kg
Selenium (Se)	2.39E-7	Kg
Nitrogen oxides (NOX)	1.06	Kg
Carbon monoxide (CO)	0.15	Kg
Particulates (b2.5 μm)	0.107	Kg

### Data Envelopment Analysis (DEA) method

The DEA method uses input and output data from each production unit to create a production boundary non-parametrically. DEA models can be product-oriented, aiming to maximize production with a given amount of inputs, or input-oriented, aiming to minimize inputs while maintaining a certain level of output [39]. The input-oriented method does not compare unit consumption with a predetermined standard or a predetermined function but evaluates the performance of Decision-Making Units (DMUs) performing similar activities under the same conditions. Instead of determining a boundary production function, this method considers the performance of farms with the highest output-to-input ratios as the efficiency frontier. In these conditions, all observed units are situated above or below the envelopment frontier. Thus, the efficiency of each production unit is measured versus the efficiency of all production units [23]. This study used the most comprehensive DEA models, i.e., the CCR model and the BCC model, to calculate the efficiency of producers in terms of energy consumption. The CCR model is based on Constant Returns to Scale (CRS), while the BCC model is based on Variable Returns to Scale (VRS). Each model has two orientations: input-oriented and output-oriented [40]. In other words, an inefficient DMU can become efficient by reducing input consumption while maintaining output levels (input-oriented) or by increasing output levels while maintaining input levels (output-oriented) [33].

The selection between input-oriented and output-oriented approaches depends on the specific characteristics of the DMUs being analyzed. For this study, an input-oriented approach was considered more suitable due to the presence of multiple inputs required to produce a single output, namely crop yield [41,42]. In DEA, technical efficiency serves as a measure of a unit’s performance, with efficiency values ranging from zero to one. A value of one indicates that the DMU operates at optimal efficiency with no potential for reducing input usage, while values below one suggest that inputs are not being utilized efficiently. Technical efficiency is defined as follows [31]:


$$Efficiency=\frac{u_{1}y_{1}^{j^{*}}+u_{2}y_{2}^{j^{*}}+\ldots +u_{N}y_{N}^{j^{*}}}{v_{1}x_{1}^{j^{*}}+u_{2}y_{2}^{j^{*}}+\ldots +u_{N}y_{N}^{j^{*}}}$$ (8)

where *u*_*1*_*, u*_*2*_*, …* are the weights assigned to the outputs *n (n = 1,2, …, N)*, and *y*_*1*_^j*^, y_*2*_^j*^, …, y_*N*_^j*^ represent the values of the outputs *n (n = 1, 2, …, N)* for the *j*th DMU. Similarly, *v*_*1*_*, v*_*2*_*, …* are the weights assigned to the inputs *m (m = 1, 2, … M)*, and $x_{1}^{j^{*}},x_{2}^{j^{*}},\ldots ,x_{3}^{j^{*}}$ are the input values *m (m = 1, 2, …, M)* for the *j*th DMU, which is studied DMU. Efficiency is typically bounded between zero and one. The linear programming model is used to optimize the production units in CRS conditions [33,43,44]:


$$\begin{array}{c} \max E_{p}=\sum {\mathrm{\backslash nolimits}}_{r=1}^{r=s}U_{r}Y_{rp}+w\\ \sum {\mathrm{\backslash nolimits}}_{i=1}^{i=m}V_{i}X_{ip}=1\\ \sum {\mathrm{\backslash nolimits}}_{r=1}^{r=s}U_{r}Y_{rj}-\sum {\mathrm{\backslash nolimits}}_{i=1}^{i=m}V_{i}{X_{i}}_{j}+w\leq o,j=1,2,...,n\\ U_{r}\geq \varepsilon ,V_{i}\geq \varepsilon ,w\;free \end{array}$$ (9)

In the above models, efficient and inefficient units are identified, and various types of technical efficiency are determined. *E*_*p*_ is the efficiency rate of the *i*th unit, *U*_*r*_ is the weight of inputs, *Y*_*rp*_ is the value of the *r*th output for DMUp, *V*_*i*_ is the weight of inputs, *X*_*ip*_ is the value of the *i*th input for DMUp, *Y*_*rj*_ is the value of the *r*th output for DMUj, *X*_*ij*_ is the value of the *i*th input for DMUj (j = 1, 2, …, n), and *s* is the number of outputs. Therefore, there were 21 DMUs for analysis. In this research, Excel 2016 and STATA software were used to calculate efficiency and analyze data.

## Results

### Analysis of energy consumption in pomegranate orchards

In this section, the quantities of inputs and outputs of pomegranate production, as well as their energy equivalents, in Kuhdasht County were estimated. Next, energy consumption indicators of crop production were calculated, and the results were analyzed. Table 5 presents the amount of inputs and outputs in the production of one hectare of pomegranate orchards in the region.

**Table 5:** Energy inputs and output for pomegranate orchards production.

Item	Unit	Quantity (ha)	Total energy equivalent (MJ)	Percentage (%)
**Inputs (unit)**				
Human labor	h	486.28	953.12	6.26
Agricultural machinery	h	11.54	723.44	4.75
Diesel fuel	L	95.19	5360.11	35.23
Manure	kg	1321.43	396.43	2.61
Phosphate (P_2_O_5_)	kg	14	174.16	1.14
Potassium	kg	26.867	299.56	1.96
Nitrogen	kg	17.45	1154.58	14.65
Herbicide	L	2.79	664.14	4.36
Electricity	kWh	293.23	3498.33	22.99
Water for irrigation	M^3^	895.28	913.19	6.01
Total input energy	MJha^-1^	–	15211.04	100
**Output**				
Pomegranate yield	kg	17028.09	32353.38	100

Based on the results, the total input and output energy to produce one hectare were 15,211.05 ±** **1,243.10 MJ and 32,353.38 ± 2,015.44 MJ, respectively. These values represent mean ± standard deviation across the 21 surveyed farms. Bootstrapped 95% confidence intervals for input and output energy were [14,780.23–15,641.87] MJha ⁻ ¹ and [31,842.11–32,864.65] MJha ⁻ ¹, indicating relatively low variability among farms. Mortazavinia and Rastegaripour (2020) [45], reported the total input energy for pomegranate production as 6,751.65 MJha ⁻ ¹ and the output energy as 13,383 MJha ⁻ ¹ in Razavi Khorasan province, Iran. The energy consumption for pomegranate production was reported as 40,064.87 MJha ⁻ ¹ in Turkey [46]. Nutritional management is an essential factor affecting fruit quantity and quality. In the region, fertilizers are spread across the orchard and mixed with the soil by tilling to a depth of 30–40 cm. The diesel fuel, electricity, and nitrogen fertilizer accounted for the highest energy consumption at 35.23%, 22.99%, and 14.65% in the pomegranate orchards of the studied area, respectively. Similar research showed that diesel fuel and nitrogen fertilizer were the most consumed energy inputs in pomegranate production, accounting for 44.15% and 29.64%, respectively [45].

According to Table 5, the descriptive statistics of input consumption for pomegranate production in the region revealed that diesel fuel was the most consumed input (95.19 ± 8.71 lha ⁻ ¹), and electricity was the second most consumed input (293.23 ± 18.56 kWhha ⁻ ¹). The high consumption rate of these inputs in the study area is associated with their low prices (owing to hidden government subsidies in the agricultural sector), the lack of mechanized infrastructure and the wear and tear of vehicles and machinery used for diesel fuel consumption. Proper consideration should be given to the consumption of these inputs.

Energy sources were further classified into direct vs. indirect and renewable vs. non-renewable, following the methodology proposed by Khanali et al (2021) [31]. Direct energy refers to energy directly used on-farm (e.g., diesel, electricity), whereas indirect energy relates to off-farm energy required for the production and transportation of inputs (e.g., fertilizers, pesticides). Renewable energy includes human labor, manure and irrigation water, while non-renewable energy includes fossil fuels and synthetic chemicals. Table 6 illustrates the distribution of direct, indirect, renewable, and non-renewable energy sources used in cultivating one hectare of pomegranate orchards. Renewable energy sources contributed 14.87% to the total energy usage, primarily from irrigation water, manure, and labor. In contrast, non-renewable energy sources accounted for a significant 85.13%. Direct energy consumption, which includes diesel fuel and electricity, dominated the energy usage with a share of 70.51%, while indirect energy sources constituted 29.49% of the total energy consumption.

**Table 6:** Energy indicators in the production of one hectare pomegranate orchards production.

Index	Unit	Amount
Energy ratio	–	2.14
Energy productivity	Kg^-1^MJ	1.12
Epecific Energy	MJkg^-1^	0.89
Net energy	MJha^-1^	17142.32
Direct energy	Percentage	70.51
Indirect energy	Percentage	29.49
Renewable energy	Percentage	14.87
Nonrenewable energy	Percentage	85.13

According to the data in Table 6, the energy efficiency ratio was 2.14, energy productivity was 1.12 kgMJ ⁻ ¹, specific energy was 0.89 MJkg ⁻ ¹, and net energy was 17,142.32 MJha ⁻ ¹. Consequently, approximately 0.89 MJ of energy is utilized for each unit of pomegranate production in these orchards. In comparison, the energy ratio and energy efficiency for mung bean cultivation in Nepal were reported to be 3.11 and 0.214 kgMJ ⁻ ¹, respectively [47]. Additionally, research indicated that the total input energy for sugar beet farming in the Cherdavel and Islamabad regions of Iran was 55,308.42 and 54,871.22 MJha ⁻ ¹, while the total energy output was approximately 940,800 and 806,400 MJha ⁻ ¹, respectively [29]. The direct energy usage accounted for 66.17% and 57.17% of the total energy consumed. For cucumber production, the renewable and non-renewable energy inputs were 2,062.31 and 51,062.7 MJha ⁻ ¹ [18], while for melon cultivation, these values were 1,738.88 and 25,416.98 MJha ⁻ ¹ [48]. Similarly, for corn production, the renewable and non-renewable energy inputs were 31,717.21 and 166,100.04 MJha ⁻ ¹, respectively [30].

### Analysis of economic indicators

The production costs in one hectare of a pomegranate orchard include expenditures on labor, pesticide application, irrigation, operational inputs, machinery use, and harvesting activities. Table 7 presents the key economic indicators of pomegranate production, including sale price, gross value, variable and fixed costs, gross revenue, and other financial metrics.

**Table 7:** The analysis of economic indicators for pomegranate orchard production.

Index	Unit	Amount
Yield	kg ha^ − 1^	17028.09
Sale price	$kg^ − 1^	0.53
Gross value	$ha^ − 1^	9081.648
Variable costs	$ha^ − 1^	1022.22
Fixed costs	$ha^ − 1^	204.44
Total cost of a production period	$ha^ − 1^	1226.67
Gross revenue	$ha^ − 1^	8059.42
Net income	$ha^ − 1^	7854.97
Benefit to cost ratio	Dimensionless	0.83

Based on this data, the gross value of production in pomegranate orchards was calculated as 9,081.64 USD ha ⁻ ¹, with fixed costs estimated at 204.44 USD ha ⁻ ¹ and variable costs at 1,022.22 USD ha ⁻ ¹. The research indicated that the largest share of total production cost was related to variable costs (83.33%), among which human labor, machinery and manure accounted for the top three expenses, respectively. Fixed costs contributed 16.67% to the total cost structure.

The BCR for pomegranate orchards was found to be 0.83, indicating that for every 1 USD invested, only 0.83 USD in return was generated. This suggests that pomegranate production in the study area is currently not economically viable under existing input cost and output price conditions. Several factors contribute to this low BCR:

- High input costs, particularly for diesel fuel, electricity, and nitrogen fertilizer, driven by subsidized pricing and inefficient use.
- Moderate yield levels, which may be increased through improved agronomic practices and better water and nutrient management.
- Low market prices received by farmers due to weak marketing channels and lack of branding or premium pricing for local pomegranate varieties.

A comparison with other crops in the region further highlights these challenges. Rashidi et al. (2024) [18], reported a BCR of 1.71 for cucumber production in nearby areas, benefiting from higher yields and more favorable market prices. Similarly, Azizpanah et al (2023) [47], observed higher BCR values for sunflower and cucumber, where efficient resource use and better access to markets led to improved returns.

According to Table 7, economic productivity was calculated to be 3.08 kg USD ⁻ ¹. This means that for every 1 USD spent on production, approximately 3.08 kg of pomegranate was produced. While this indicates a relatively efficient conversion of inputs into output from an energy-productivity standpoint, it does not compensate for the overall negative return on investment when considering current cost structures and market prices.

These findings suggest the need for targeted interventions such as input optimization, improved post-harvest handling and market access improvements to enhance the profitability of pomegranate farming in the region.

### The results of optimization by DEA

Table 8 shows the analysis of pomegranate orchards using the input-oriented CCR model through DEA. The average technical efficiency obtained in inefficient orchards using the input method was 98.96%, meaning that by utilizing 98.96% of the current inputs while maintaining the same output level, inefficient units can reach the efficiency frontier. This implies an average potential input saving of 1.035% among inefficient units.

**Table 8:** Evaluation of pomegranate orchards by CCR input oriented model.

DMU	Rank	CRS (%)	VRS (%)	ES (%)	Unit samples and decision variable coefficients	Return to sclae
dmu:1	1	100	100	100	–	Constant
dmu:2	1	100	100	100	–	Constant
dmu:3	1	99.34	100	99.48	5 (37.53)	Increasing
dmu:4	1	100	100	100	–	Constant
dmu:5	17	98.65	100	98.89	1 (27.56), 2 (32.73), 6 (29.27)	Increasing
dmu:6	1	100	100	100	–	Constant
dmu:7	19	99.94	99.97	99.98	2 (1.17), 4 (33.7), 15 (38.17), 19 (13.99), 21 (12.9)	Increasing
dmu:8	1	100	100	100	–	Constant
dmu:9	1	100	100	100	–	Constant
dmu:10	1	100	100	100	–	Constant
dmu:11	1	96.02	100	97.05	9 (27.63), 13 (25.82)	Increasing
dmu:12	20	94.14	97.84	96.22	1 (4.99), 2 (29.11), 19(4.51), 20 (59.48)	Increasing
dmu:13	18	97.71	100	98.85	2 (88.13), 9 (2.86), 20 (1.98)	Increasing
dmu:14	1	100	100	100	–	Constant
dmu:15	1	100	100	100	–	Constant
dmu:16	1	100	100	100	–	Constant
dmu:17	21	92.41	93.01	99.35	2(6.64), 15(29.02),18(4.89), 19(20.53), 20(29.23), 21(9.37)	Increasing
dmu:18	1	100	100	100	–	Constant
dmu:19	1	100	100	100	–	Constant
dmu:20	1	100	100	100	–	Constant
dmu:21	1	100	100	100	–	Constant
Average	–	98.96	99.56	99.39	–	–
Sum	–	2078.25	2090.83	2087.29	–	–

According to Table 8, orchards 1, 2, 4, 6, 8, 9, 10, 14, 15, 16, 18, 19, 20, and 21 were technically efficient. Out of the 21 pomegranate orchards, 14 units demonstrated 100% technical efficiency, while 7 units exhibited varying degrees of technical inefficiency. In other words, 66.68% of the units were fully technically efficient, and 33.32% were technically inefficient.

The 96.02% efficiency score for unit 11 indicates that this orchard must reduce 3.98% of all its production inputs without reducing output to achieve full efficiency. Since its reference set includes orchards 9 and 13, it is suggested that unit 11 could improve its performance by benchmarking best practices from these efficient units. Specifically, it would benefit from adopting 27.63% of the resource allocation strategies used by orchard 9 and 25.82% from orchard 13, which are considered efficient models in terms of resource use.

Based on the CRS model, pomegranate orchards 3, 5, 11, and 13 showed partial efficiency. While their net technical efficiency under CRS is equal to one, their overall efficiency is less than one due to scale or managerial inefficiencies identified under the VRS model. The RTS status is determined by the lambda (λ) values: if λ < 0, RTS is increasing; if λ > 0, RTS is decreasing; and if λ = 0, RTS is constant.

According to Table 8, orchards 1, 2, 4, 6, 8, 9, 10, 14, 15, 16, 18, 19, 20, and 21 exhibit constant returns to scale (i.e., optimal size), whereas the remaining orchards show increasing returns to scale, suggesting that they could improve efficiency by scaling up operations.

Saved energy of each input in pomegranate orchards using the VRS and CRS models:

Table 8 shows that an average of 1891 MJ ha ⁻ ¹ of energy can be saved in the studied orchards using the VRS model, accounting for approximately 12.43% of the total input energy per hectare. Under this model, the highest average energy savings were observed in irrigation water, manure, labor, electricity, and potassium fertilizer, respectively.

Using the CRS model, the average energy savings were 786.37 MJ ha ⁻ ¹, which is lower than the VRS estimate (Table 9). However, similar trends were observed, with the most significant savings related to manure, irrigation water, labor, electricity, and potassium fertilizer. These findings suggest that improved management practices can lead to meaningful energy savings under both models.

**Table 9:** Amounts of stored energy of the inputs in pomegranate orchards using VRS and CRS models (MJha^-1^).

	Analysis model	Potassium	Nitrogen	Phosphorus	Agricultural machinerry	Water for irrigation	Human labor	Manure	Electricity	Diesel fuel	Poison	Total
Average	VRS	44.48	24.77	15.08	0.51	286.7	216.93	1234.90	59.99	6.09	1.48	1891
Average	CRS	39.15	21.93	14.74	0.43	201.09	214.49	232.79	56.47	4.09	1.13	786.37

### Environmental indicators of pomegranate production

The environmental impacts of producing one ton of pomegranate were assessed using the LCA method under both conventional and optimized input usage scenarios. Table 10 presents the midpoint-level environmental impact indicators for pomegranate production. As shown, if inefficient orchards adopt optimized input consumption patterns and reach the efficiency frontier, the environmental impacts across 15 categories are projected to decrease by 1.69–37.12%.

**Table 10:** The amount of the environmental impact of pomegranate production (midpoints).

Indicator	Unite	Value		
		Conventional pomegranate	Optimized pomegranate	Percentage(%)
Carcinogens	kg C_2_H_3_Cl eq	0.231	0.215	6.47
Non-carcinogens	kg C_2_H_3_Cl eq	1.761	1.262	28.31
Respiratory inorganics	kg PM2.5 eq	19.17060866	12.053	37.12
Ionizing radiation	Bq C-14 eq	27569.83371	23679.725	14.11
Ozone layer depletion	kg CFC-11 eq	6.28214E-07	5.18574E-07	17.45
Respiratory organics	kg C_2_H_4_ eq	0.0228	0.0201	11.74
Aquatic ecotoxicity	kg TEG water	3007.903	2956.968	1.69
Terrestrial ecotoxicity	kg TEG soil	11649.695	11443.082	1.77
Terrestrial acid/nutri	kg SO_2_ eq	1.101	1.073	2.5
Land occupation	m2org.arable	0.213	0.1951	8.711
Aquatic acidification	kg SO_2_ eq	0.168	0.148	11.93
Aquatic eutrophication	kg PO_4_ P-lim	0.0196	0.0166	15.13
Global warming	kg CO_2_ eq	40.563	35.975	11.31
Non-renewable energy	MJ primary	355.422	299.608	15.71
Mineral extraction	MJ surplus	0.726	0.584	19.56

The smallest reduction (**1.69%**) was observed for the aquatic ecotoxicity indicator (kg TEG water), while the largest reduction (37.12%) occurred in the respiratory inorganics category (kg PM₂.₅ eq). Other notable reductions included:

- Non-carcinogens: 28.31%
- Mineral extraction: 19.56%
- Ozone layer depletion (kg CFC-11 eq): 17.45%
- Non-renewable energy use (MJ primary): 15.71%

The significant reduction in ozone layer depletion indicates that optimizing inputs can reduce emissions of substances harmful to stratospheric ozone. Ozone thinning increases UV radiation exposure, which negatively affects human health, ecosystems, and materials.

These endpoint-level results show that human health is the most significantly affected category under current practices, followed by ecosystem quality, resources, and climate change. Key contributors to human health impacts include carcinogens, non-carcinogens, respiratory inorganics, ionizing radiation, and ozone-depleting substances. By identifying these factors, targeted improvements in input management can yield substantial benefits.

A sensitivity analysis was conducted to evaluate the influence of variability in key inputs such as diesel fuel consumption, nitrogen fertilizer application rates, and electricity use on the overall impact categories. Results indicate that a ± 10% variation in diesel consumption leads to changes of up to ±4.5% in global warming potential and ±6.2% in respiratory inorganics. Confidence intervals have been added to support the robustness of the main findings, particularly for the 37.12% reduction in respiratory inorganics (95% CI: 34.6–39.8%).

All units and abbreviations used in this study are now clearly defined at first mention [49 50]:

- TEG = Toxicity Equivalent Quantity
- TEG water = kg TEG water (aquatic toxicity equivalent)
- TEG soil = kg TEG soil (terrestrial toxicity equivalent)
- DALY = Disability Adjusted Life Year
- PDF·m²·yr = Potentially Disappeared Fraction per square meter per year

**Fig 2:**
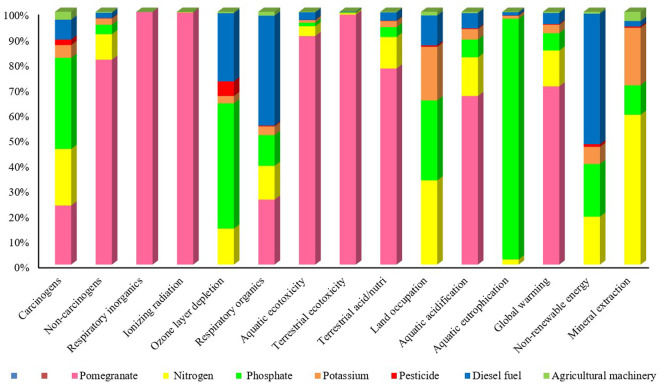
The evaluation of environmental indicators in 15 categories of environmental effects after optimization of inefficient units.

Fig 2 illustrates the evaluation of environmental impacts across 15 categories after optimization of inefficient units. Despite improvements, pomegranate production remains a significant source of pollution, especially in the ozone layer depletion category, where phosphate fertilizers, diesel fuel, and nitrogen fertilizers were identified as major contributors. In the global warming category, direct emissions from diesel combustion and nitrogen fertilizers dominated the impact. Similarly, aquatic acidification and respiratory organics were strongly influenced by chemical fertilizer and diesel use.

These findings align with previous studies, such as those on cucumber production in Dareshahr, Iran [18], which highlighted the importance of nitrogen fertilizer in driving environmental impacts. Research by Rafiee et al (2016) [51], also supports our observation that electricity and animal manure contribute significantly to respiratory inorganics, while Rajaeifar et al (2015) [52], found urea and phosphate fertilizers to be dominant in terrestrial ecotoxicity.

The normalized aggregated results of the environmental indicators in the pomegranate production system, i.e., the impact indicators in the four mid-point effect indicators of human health, ecosystem quality, climate change, and resources, are presented in Table 11.

**Table 11:** The amount of the environmental impact pomegranate production (end-points).

Indicator	Unite	Value (Total)	
		Conventional pomegranate	Optimized pomegranate
Human health	DALY	356.006	300.335
Ecosystem quality	PDF m^2^ yr	95.649	86.021
Climate change	kg CO_2_ eq	40.563	37.975
Resources	MJ primary	70.048	61.013

Rasoolizadeh et al (2022) [53], evaluated the eco-efficiency of selected tropical fruit production systems in Iran using LCA and DEA, highlighting the importance of such analyses for transitioning towards sustainable agriculture. The combined use of LCA and DEA allows for a comprehensive assessment of environmental efficiency, as demonstrated by Ewertowska et al (2017) [54], who used Monte Carlo simulation to quantify environmental efficiencies under uncertainty. Yang et al (2022) [55], also utilized a combined life cycle and data envelopment analysis to reconcile productivity, profitability, and sustainability of small-holder sugarcane farms, demonstrating the broad applicability of this approach. Abdi et al (2023) [49], illustrates a multi-step process for evaluating environmental impacts and eco-efficiency related to Decision-Making Units (DMUs) using LCA and DEA.

## Conclusions

This research evaluated energy consumption patterns, environmental impact indicators, and conducted an economic analysis of pomegranate production in the Kuhdasht region of Lorestan province. Estimating efficiency improvement indicators and increasing productivity are among the main goals of agricultural production units. The results regarding technical efficiency and optimal use of production inputs showed that environmental effects can be significantly reduced without compromising crop yield, simply by improving resource-use efficiency. Following the optimization of input consumption through DEA-based modeling, the lowest reduction across environmental impact categories was observed for the aquatic ecotoxicity indicator (1.69%), followed by the terrestrial ecotoxicity indicator (1.77%). In contrast, the highest reduction was recorded for the respiratory inorganics impact indicator (37.12%), followed by the non-carcinogens indicator (28.31%). These findings suggest that optimizing input usage can have a disproportionately large effect on reducing air pollution and human health risks. The LCA results revealed that pomegranate production in the study area had the greatest impact on the human health damage category, primarily due to emissions contributing to respiratory inorganics and carcinogenic effects. Across all impact categories, phosphate fertilizer, diesel fuel and nitrogen fertilizer were identified as the main contributors to both environmental burdens and production costs. In general, when comparing conventional and optimized cultivation practices, it was evident that optimized cultivation mode resulted in lower environmental impacts across most indicators. Notably, the demand potential for non-renewable energy was calculated as 355.42 MJ kg ⁻ ¹ under conventional practices, which decreased to 299.6 MJ kg ⁻ ¹ in the optimized scenario. According to the results, and considering the necessity of pomegranate production to meet domestic demand and its significant export value, it is recommended that stakeholders implement several key interventions:

- Reform irrigation systems in orchards to reduce water consumption.
- Encourage farmers to conduct regular soil tests to determine optimal chemical fertilization rates.
- Improve farmer awareness and training regarding the efficient use of agrochemicals.

## Supporting information

S1 Data Data. (XLSX)
